# First whole-genome assembly of the Galápagos Petrel (*Pterodroma phaeopygia*) using Oxford Nanopore sequencing to advance conservation genomics in a critically endangered seabird

**DOI:** 10.1093/g3journal/jkag004

**Published:** 2026-01-27

**Authors:** Isabella R Sessi, James B Henderson, Jessica A Martin, Alice Skehel, Gabriela Pozo, Jonathan A Guillén Alcides, Vera de Ferran, John P Dumbacher, Jaime A Chaves

**Affiliations:** Department of Biology, San Francisco State University, San Francisco, CA 94132, United States; Institute for Biodiversity Science and Sustainability, California Academy of Sciences, San Francisco, CA 94118, United States; Institute for Biodiversity Science and Sustainability, California Academy of Sciences, San Francisco, CA 94118, United States; Department of Biology, San Francisco State University, San Francisco, CA 94132, United States; Institute for Biodiversity Science and Sustainability, California Academy of Sciences, San Francisco, CA 94118, United States; Faculty of Science and Engineering, University of the Sunshine Coast, Sippy Downs, Queensland 4556, Australia; Laboratorio de Biotecnología Vegetal, Colegio de Ciencias Biológicas y Ambientales, Universidad San Francisco de Quito USFQ, Quito 170901, Ecuador; Alsacio Northia y Angel Serrano, San Cristóbal, Islas Galápagos 200101, Ecuador; Department of Biology, San Francisco State University, San Francisco, CA 94132, United States; Institute for Biodiversity Science and Sustainability, California Academy of Sciences, San Francisco, CA 94118, United States; Department of Biology, San Francisco State University, San Francisco, CA 94132, United States; Institute for Biodiversity Science and Sustainability, California Academy of Sciences, San Francisco, CA 94118, United States; Department of Biology, San Francisco State University, San Francisco, CA 94132, United States; Colegio de Ciencias Biológicas y Ambientales, Universidad San Francisco de Quito, Quito 170901, Ecuador; Galapagos Science Center, Universidad San Francisco de Quito USFQ, Islas Galápagos 200101, Ecuador

**Keywords:** long reads, Galápagos Islands, endangered species, invasive species, genome assembly

## Abstract

The Galápagos Petrel (*Pterodroma phaeopygia*) is a critically endangered procellariiform seabird endemic to the Galápagos Islands. Once abundant, its populations have sharply declined due to invasive predators, habitat degradation, and destruction of nest burrows. Although the species is distributed across several islands, the demographics of each population and their genetic relationships are poorly understood. To facilitate future studies of population structure and connectivity, we present the first high-quality reference genome for the Galápagos Petrel. The genome was assembled solely from ultra-long Oxford Nanopore sequence data collected from an adult female sampled on San Cristóbal Island. Sequencing was performed at the Galapagos Science Center, building local capacity for the generation of genomic data in remote regions. The final nuclear genome assembly spans 1.35 Gb in length, with average coverage of 36.07×, scaffold N50 of 74.2 Mb, and a BUSCO completeness of 99.95%. The genome comprises 41 pseudo-chromosomes, with 23 spanning from telomere to telomere and 16, including W and Z chromosomes, containing a single telomere. Chromosomal-level scaffolding by reference was performed using the genome of Cory's Shearwater (*Calonectris borealis*), a closely related species. This reference genome provides a foundational tool for comparative genomics, conservation biology, and functional studies of island-endemic avifauna, and demonstrates that recent advances in basecalling and error correction now enable Oxford Nanopore Technologies-only datasets to achieve assemblies comparable in quality to those generated using short-read or PacBio HiFi data. It will also facilitate future efforts to characterize genetic diversity, structural variation, and adaptive responses in this critically endangered species.

## Teaser

This study presents the first whole-genome assembly of the critically endangered Galápagos petrel (Pterodroma phaeopygia), a seabird endemic to the Galápagos islands. Using Oxford Nanopore ultra-long sequencing and scaffolding with a related species, Sessi et al. produced a high-quality, chromosomal-level genome - the first for Pterodroma and only the second for the Procellariidae family. This assembly enables new research into sex-linked genes, population structure, and inbreeding among Galápagos petrels. This resource fills a major taxonomic gap and offers essential genomic tools for future conservation efforts and seabird evolutionary studies. Conservation biologists, geneticists, and evolutionary researchers will benefit from this foundational genomic resource.

## Introduction

The Galápagos Islands have long been renowned as a unique biodiversity hotspot, home to thousands of endemic plant and animal species (Kricher and Loughlin 2022), many of which have small population sizes and rely on a healthy environment to survive (Fernández-Palacios et al. 2021). Since the arrival of humans in the 1500s, hundreds of invasive species have been introduced to the islands, including livestock, rats, cats, dogs, and plants (Phillips et al. 2012; Causton et al. 2015). The introduction of invasive species, compounded with increased human hunting, fishing, and habitat destruction, has reduced native species populations, lowering genetic diversity and pushing some Galápagos endemic species to the brink of extinction (Phillips et al. 2012; Chaves 2018).

Galápagos Petrels (*Pterodroma phaeopygia*), a critically endangered Galápagos endemic, have a large range outside the archipelago when not breeding, extending across the eastern Pacific from the coasts of Mexico to Peru and westward in the tropics to French Polynesia (Granizo 2002; BirdLife International 2018). When breeding, Galápagos Petrels return to nesting colonies on five islands: Santa Cruz, San Cristóbal, Floreana, Santiago, and Isabela (Cruz-Delgado et al. 2010, Fig. 1a). Each of these populations is demographically isolated from the others due to nest site fidelity; that is, individual petrels born on a given island return to the same island to breed each year (Cruz and Cruz 1990).

**Fig. 1.**
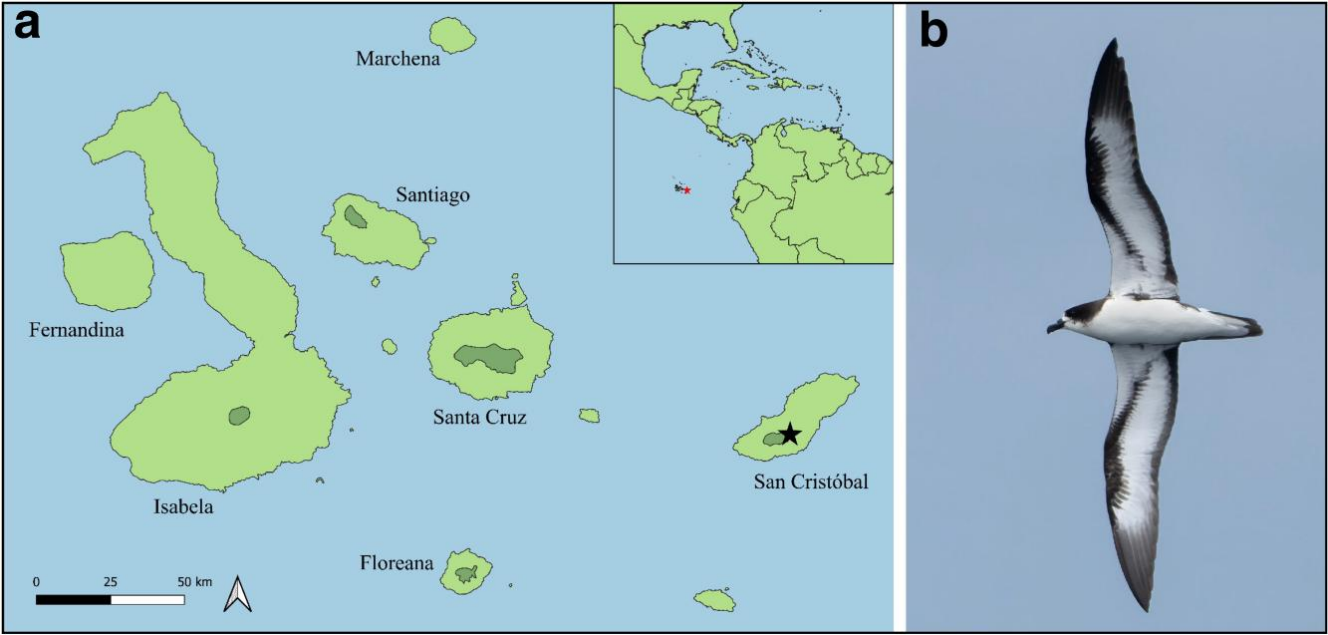
Galápagos Islands and Galápagos Petrel. a) Map of the Galápagos Islands showing known Galápagos Petrel (*P. phaeopygia*) breeding colonies (dark shaded areas) across five islands: San Cristóbal, Santa Cruz, Santiago, Floreana, and Isabela. The genome sampling location at La Comuna, San Cristóbal Island, is indicated by a star (modified from IUCN Red List spatial data; BirdLife International and Handbook of the Birds of the World, 2017). b) A Galápagos Petrel (Photo: Birding Experience).

Galápagos Petrels face ongoing population declines from human-induced threats (Zurita-Arthos et al. 2023). As a burrowing species, they are particularly sensitive to invasive plants that grow over nest entrances and make nesting territories inhospitable (Zurita-Arthos et al. 2023). They fall prey to invasive rats, cats, dogs, and feral pigs, which raid nests, depredating on eggs, young (Tapia-Jaramillo et al. 2025), and adult petrels (Harris 1970; Duffy 1984). More than 90% of the petrels' nesting habitat is located on privately owned land, leading to increased habitat loss due to agricultural development (Cruz-Delgado et al. 2010; Zurita-Arthos et al. 2023). Grazing livestock in such areas trample and ruin petrel nesting areas (Duffy 1984; Cruz-Delgado et al. 2010). These anthropogenic factors have led to low breeding success, and Galápagos Petrel populations have declined dramatically, leading to their listing as a critically endangered species by the IUCN (Tomkins 1985; Cruz-Delgado et al. 2010; BirdLife International 2018).

Previous studies on the genetics of the Galápagos Petrel have been limited in scope and primarily focused on a few microsatellites, leaving much unknown about the genetic status of this critically endangered species (Browne et al. 1997; Friesen et al. 2006). A whole-genome assembly for this species will enable detailed population genomics studies, key tools for assessment, monitoring, and management of the species (Brito and Edwards 2009).

Recent studies have highlighted the importance of genomic data in advancing conservation efforts (Romanov et al. 2009; Theissinger et al. 2023; Hogg 2024), but these often depend on access to substantial technological infrastructure. The improvement of low-cost portable sequencing platforms offers a solution that is well-suited for use in remote and logistically challenging environments (Watsa et al. 2020) such as the Galápagos Islands. In these places, we cannot rely on commercial high-throughput sequencing facilities for library preparation and sequencing. Moreover, international movement of biological samples is heavily restricted for endangered species protected under the United States Endangered Species Act (ESA) and the Ecuadorian implementation of the Nagoya Protocol (Secretariat of the Convention on Biological Diversity 2010). Sequencing on the island served two key purposes: (i) building local capacity and supporting local conservation genomics efforts, and (ii) abiding by the regulations of protected species.

Here we present the first assembled whole genome of the critically endangered Galápagos Petrel (*P. phaeopigia*), sequenced in the Galápagos, including both W and Z sex avian chromosomes.

## Methods and materials

### Specimen collection

Approximately 50 µl of blood was drawn from the brachial vein of an adult female Galápagos Petrel, using a 27-gauge needle and a 1 ml syringe on the 2024 July 2 in La Comuna, San Cristóbal Island (0°53′04.5″S, 89°27′54.9″W). Sample collection was conducted in accordance with the regulations of the authorities and guidelines for the use of birds in field research. The sample was preserved in NAP buffer (Camacho-Sanchez et al. 2013) at the collection site and stored at −80 °C at the Galapagos Science Center (GSC) until DNA extraction.

### DNA isolation and sequencing

High molecular weight (HMW) DNA was extracted using the Monarch UHMW DNA Extraction Kit for Cells and Blood (NEB #T3010, New England Biolabs, Ipswich, MA) following the manufacturer's protocol. DNA concentration was measured using Qubit Fluorometric Quantitation (Thermo Fisher Scientific). A library was prepared with the Oxford Nanopore Technologies (ONT) Ultra-Long DNA Sequencing Kit (SQK-ULK114), and sequenced on three PromethION 2 flow cells (FLO-PRO114M) using the PromethION 2 Solo (P2 Solo) platform. A second library was prepared from the same sample in January 2025 during a follow-up field trip, using the same protocols and sequenced using two flow cells.

Both sequencing runs were conducted on-site at the GSC using a portable Windows DELL laptop (Supplementary Section 2.1) connected to the P2 Solo device. Raw sequence signal was saved to the laptop in POD5 files, a streaming format for direct sequencing instrument output (http://github.com/nanoporetech/pod5-file-format). ONT was selected due to its portable sequencing capabilities and for its ability to generate ultra-long reads for assembling through complex and repetitive genomic regions with newer R10 flow-cell Kit 14 chemistry and improved software (https://github.com/nanoporetech/dorado) (Stanojević et al. 2024; Cheng et al. 2025), producing reads with error profiles suitable for current long-read assembly programs.

### Basecalling, adapter trimming, and error correction

Each of the two runs were independently basecalled and adapter-trimmed as follows. POD5 files, copied from the laptop computer used during sequencing at the GSC, were basecalled on a GPU-enabled computer using the Dorado v0.7.4 basecaller in super-accurate mode (SUP) via MinKNOW Core v6.0.8. This produced multiple 4K fastq files, which were combined into complete datasets for each of the two runs.

Adapters were trimmed using Porechop_ABI v0.5.1 (Bonenfant et al. 2023) with the –ab_initio argument. The trimmed reads were then error corrected with Dorado v0.9.1 using arguments correct –cpu -t 96 on an HPC Linux server, producing fasta-formatted corrected reads.

The two versions of Dorado were used due to software availability on separate systems: basecalling was performed at the California Academy of Sciences (CAS) using MinKNOW, which at the time incorporated Dorado v0.7.4, while read correction was performed later on our HPC server using the more recent Dorado v0.9.1.

Read statistics and quality metrics for trimmed-only reads, Hifiasm corrected reads, as well as a subset of metrics for Dorado corrected reads, were calculated using NanoPlot (De Coster et al. 2018) and a custom script (seq_summary_qscore_lens.sh, see Code Availability).

### Genome size estimate

Initial read coverage, genome size, and heterozygosity levels were estimated using GeneScopeFK (https://github.com/thegenemyers/GENESCOPE.FK), with kmer sizes 21, 25, 27. This is a command line implementation of GenomeScope2 (Ranallo-Benavidez et al. 2020) with identical results, built-in kmer histogram production, and plot figure file creation. Results can be found in Supplementary Section 1.1 (Supplementary Figs. 1 and 2).

### Genome assembly and scaffolding

**Fig. 2.**
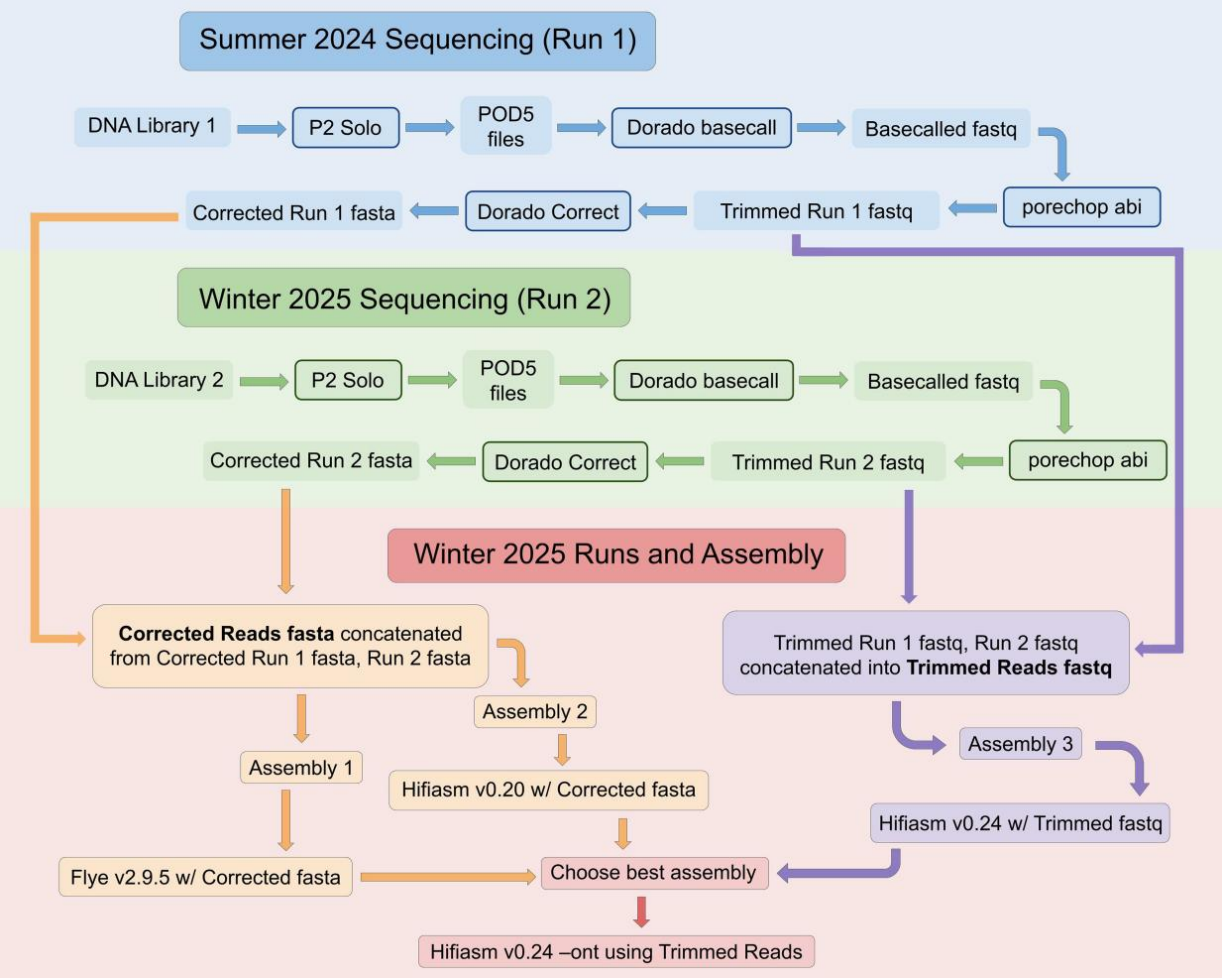
Overview of the sequencing and assembly workflow for the genome of the Galápagos Petrel (*P. phaeopygia*). Two Oxford Nanopore libraries were generated and processed independently. Raw reads were collected as POD5 files from P2 Solo flow cells and basecalled with Dorado, producing basecalled FASTQ files. Adapter sequences were trimmed using Porechop_ABI, yielding trimmed FASTQ reads. At this stage, the two libraries were also merged and assembled using Hifiasm v0.24 –ont to generate an initial assembly for comparison. Separately, each library's trimmed reads were error-corrected with Dorado Correct, producing corrected FASTA files. The corrected reads from both libraries were concatenated and assembled with Flye v2.9.5 and Hifiasm v0.20, respectively. All three assemblies were evaluated for quality, and the best-performing assembly was selected as the final reference genome.

Three assemblies were produced for comparison. The basecalled and trimmed files from the two runs were concatenated into a single fastq and used as input to Hifiasm v0.24 (Cheng et al. 2021, 2025), which was released during the preparation of this assembly. This version of Hifiasm includes built-in error correction optimized for ONT simplex R10 reads. Separately, the trimmed reads from each run were also error-corrected using Dorado v0.9.1. These two sets of Dorado corrected reads were used as input for the Flye assembler and for a previous version of Hifiasm, v0.20. (Fig. 2).

These three assemblies were then evaluated: (i) Flye v2.9.5, using error-corrected reads input as –nano-corr along with the –scaffold option, (ii) Hifiasm v0.20, run with the same corrected reads used for Flye; and (iii) Hifiasm v0.24, using trimmed reads with the –ont option.

All three assemblies were checked for adapter and other contamination using the NCBI Foreign Contamination Screen tools (https://github.com/ncbi/fcs), FCS-adaptor, and FCS-GX (Astashyn et al. 2024), against taxonomy ID 53680, and evaluated for standard metrics including N50, L50, using in-house modified script asmstats.pl (see Code Availability) and for genome completeness via Compleasm v0.2.6 (Huang and Li 2023), miniprot (Li 2023), and BUSCO v5.4.7 (Simão et al. 2015), MetaEuk (Karin et al. 2020), with the 8,338 ortholog aves_odb10 lineage database. Statistics from asmstats, seqtk telo (https://github.com/lh3/seqtk), and combined Compleasm, BUSCO output were compiled after major pipeline stages to assess modifications. Assembly statistics (Table 1) led us to select Hifiasm v0.24 as the assembler with which to proceed.

**Table 1.** Comparison of the three contig-level assemblies for *P. phaeopygia*. Bolded values indicate the best or tied for the best category; italics indicate the second best.

Metric	Hifiasm v0.24	Flye v2.9.5	Hifiasm v0.20
Number of contigs	**109**	1,038	*918*
Total contig length	1.408G	1.31G	1.446G
Longest contig	**207.299M**	*104.336M*	26.217M
N50/L50	**36.27M**/**8**	*20.93M/17*	7.29M/64
N90/L90	**8.79M**/**39**	*2.14M/93*	1.17/234
auN50	**69.18M**	*32.81M*	8.19M
BUSCO counts, *n*:8,338	**C:8334**, **D:58, F:3, M:1**	*C:8333*, **D:58**, *F:4*, **M:1**	C:8329, D:623, F:7, *M:2*
BUSCO percentages, *n*:8,338	**C:99.95%**, **F:0.04%**, **M:0.01%**	C:99.94%, F:0.05%, **M:0.01%**	C:99.89%, F:0.08%, M:0.02%

Contaminants, primarily mitochondrial, with minimal viral or bacterial presence, were identified and filtered by assembling the mitochondrial genome (see Mitochondrial assembly and annotation section) and comparing the resultant complete mitochondrion against the nuclear genome via blastn (Altschul et al. 1990; Camacho et al. 2009). To avoid removing nuclear-integrated mitochondrial sequences (NUMTs), a minimum 80% read length mapping was required to identify a read as a mitochondrial contaminant.

After removing reads flagged as mitochondrial or foreign contamination, the selected assembler, Hifiasm v0.24, was run with this cleaned input to generate a consensus primary contig-level genome. Assembly output is in gfa format and not the fasta format required by downstream processing (https://gfa-spec.github.io/GFA-spec), so we converted the gfa to fasta and extracted circular contigs to a separate file with a custom script using awk (see Code Availability). Redundant haplotigs and low-confidence contigs were removed using purge_dups v1.2.5 (Guan et al. 2020), and the resulting purged fasta file was used for all subsequent analyses. Additionally, two contigs identified in BUSCO analysis as duplicates were excluded.

We performed reference-based scaffolding (Dunn 2024; Alvarez-Costes et al. 2025; Jorquera et al. 2025) with RagTag v2.28 (Alonge et al. 2022) to provide contig groupings, locations, orientations, and confidence intervals. As a scaffolding reference, we used the NCBI genome GCA_964195595.2 Cory's Shearwater (*Calonectris borealis*), which was chosen as the closest procellariiform relative with a high-quality chromosome-level genome assembly. The hap1 assembly, not NCBI RefSeq hap2, was chosen since hap1 includes W, Z, as well as the 39 numerically identified autosomal pseudo-chromosomes. Single copy gene presence/absence was cross-validated against the Cory's Shearwater information using BUSCO Aves lineage orthologs. Gap filling of the scaffolded assembly was conducted using GapFiller v1.2.1 within the quarTeT toolkit (Lin et al. 2023) with the reference scaffolded assembly and Hifiasm corrected reads as input. Records in homology with reference pseudo-chromosomes have names prefixed *Pphae* and numbered as the *C. borealis* homolog; the rest were prefixed *unloc* to indicate unlocalized sequence to any of the *C. borealis* pseudo-chromosomes (bPteroPhaeo_1.0.fasta).

### Repeat analysis

Before gene annotation, *de novo* repeats were identified using RepeatModeler v2.0.1 (Flynn et al. 2020), which uses RECON (Bao and Eddy 2002), RepeatScout (Price et al. 2005), and LtrHarvest/Ltr_retriever (Ellinghaus et al. 2008; Ou and Jiang 2018). Repeat models identified through *de novo* analysis of the assembly were combined with curated Aves models from Dfam 3.8 (https://dfam.org/releases/Dfam_3.8) and used as input for RepeatMasker v4.0.9 (Smit et al. 2015). This process generated a table summarizing repeat types and lengths, as well as a repeat-masked (soft-masked) version of the assembly, which was subsequently used for gene model annotation.

### Genome annotation

We performed ab initio gene model annotation using BRAKER3 v3.0.8 (Gabriel et al. 2024) run in EP mode (clade-level database of protein families for modeling, no species RNAseq) with the soft-masked genome assembly and GeneMark-EP+ (Brůna et al. 2021 and AUGUSTUS (Hoff and Stanke 2019) employing the vertebrate protein database from OrthoDB v11 (Kuznetsov et al. 2023). We added the argument –busco_lineage=aves so BRAKER3 invokes Compleasm with this lineage and refines results with its output. The gff3 annotation, coding sequence DNA, and protein sequence amino acid files were then used as input for further refinement and functional annotation.

Gene models without start and stop codons were removed from the BRAKER3 output files, as were models contained completely within another. Protein domains were identified in the amino acid (AA) sequences by running InterProScan v5.72-103.0 (Jones et al. 2014). The sequences, DNA or AA as appropriate, were also searched against GenBank databases (downloaded 2025 January 11) using blastn with nt database, blastp with uniprot_sprot, and diamond blastp (Buchfink et al. 2021) with TrEMBL and nr databases. Diamond blastp was also used to search OrthoDB v11 vertebrate protein AA sequences. Sequences were additionally aligned to the eggNOG v5.0.2 database with eggNOG-mapper v2.1.11 (Cantalapiedra et al. 2021). Protein domain IDs and Gene Ontology terms from the InterProScan output were added to the gff3 and sequence files for each gene model as was the functional annotation description from the lowest eValue for each gene from the blast or eggNOG searches when one or more were found with eValue lower than 1e−10.

Gene models were evaluated with BUSCO in protein mode, lineage aves_odb10, and with OMArk v2.0.3 (Nevers et al. 2025) using the OMA LUCA.h5 database to assess proteome completeness with Hierarchical Orthologous Groups (HOGs). Statistics were generated from the annotation-augmented gff3 file with a custom script (basic_gff_stats.sh, see Code Availability).

### Mitochondrial assembly and annotation

HiFiMiTie v0.08 (https://github.com/calacademy-research/HiFiMiTie), a long-read-based program for mitochondrial assembly, was used with reads previously corrected in a preliminary Hifiasm v0.24 run. This approach facilitated both the removal of mitochondrial contaminants from the nuclear genome assembly input and the generation of a complete mitochondrial genome for this species. Reads matching Aves entries in the NCBI mitochondrial database (downloaded 2025 January 11) with 50% or greater sequence coverage from a blast search were used to construct a consensus mitochondrion, its annotation, and analysis of the *control region* heteroplasmy. The Aves taxonomy was also used to define the canonical starting tRNA for the sequence and to select the appropriate mitochondrial genetic code. MITOS2 (Bernt et al. 2013; Donath et al. 2019), running locally, was used to confirm results produced by the pipeline. A circular mitochondrial depiction was created with Geneious Prime 2025.1.2 (www.geneious.com) by importing the fasta sequence and gff annotation, setting the sequence as circular, and then saving the displayed figure with the File > Save as image file option.

### Genome comparison

To evaluate the quality and contiguity of the Galápagos Petrel genome assembly, we compared our results with published genomes from three other species within the Procellariidae family: *C. borealis* (Cory's Shearwater, GCA_964195595.2), *Pelecanoides urinatrix* (Common diving petrel, GCA_013400755.1), and *Ardenna gravis* (Great shearwater, GCA_045784155.1) (Table 2).

**Table 2.** Comparison between Galápagos Petrel genome and other published genomes in the Procellariidae family.

Metric	Galápagos Petrel (*P. phaeopygia*)	Cory's Shearwater (*C. borealis*)	Common Diving Petrel (*P. urinatrix*)	Great Shearwater (*A. gravis*)
Date	2025	2024 July 23	2020 July 10	2024 December 6
Genome size	1.35 Gb	1.37	1.2 Gb	1.3 Gb
Genome coverage	36.07×	66×	81×	185×
Number of scaffolds	44	354	51,720	29,830
Longest scaffold	221,226,871	221,110,000	2,184,048	54,453,807
Scaffold N50/L50	74.2 Mb/5	86.0/5	250.3 kb/1,346	11.6 Mb/30
Scaffold N90/L90	11,894,639/24	9,973,482/25	40,369/5,737	1,752,205/126
Number of contigs	56	879	71,017	41,167
Longest contig	221,226,871	15,265,181	8,42,363	1,821,396
Contig N50/L50	46,848,837/7	4,142,416/99	93.9 kb/3,517	212 kb/1,619
Contig N90/L90	10,797,075/30	1,128,301/348	17,709/14,337	40,045/6,645
BUSCO scores (*n* = 8,338)	C:8334, D:58, F:3, M:1	C:8333, D:95, F:3, M:2	C:8030, D:17, F:219, M:89	C:8293, D:280, F:33, M:12
BUSCO percentage	C:99.95%, F:0.04%, M:0.01%	C:99.94%, F:0.04%, M:0.02%	C:96.31%, F:2.62%, M:1.07%	C:99.46%, F:0.40%, M:0.14%
Assembly level	Chromosome	Chromosome	Scaffold	Scaffold
Number of (Pseudo) chromosomes	41	41	…	…
Sequencing technology	ONT Ultra-long/P2 Solo	PacBio, Arima2	Illumina HiSeq	DIP-SEQ
Assembly program	Hifiasm v0.24	Hifiasm, YaHS	SOAPdenovo v. 2.04	supernova-2.0.1 v. 01:00:00

### BUSCO phylogeny

**Fig. 3.**
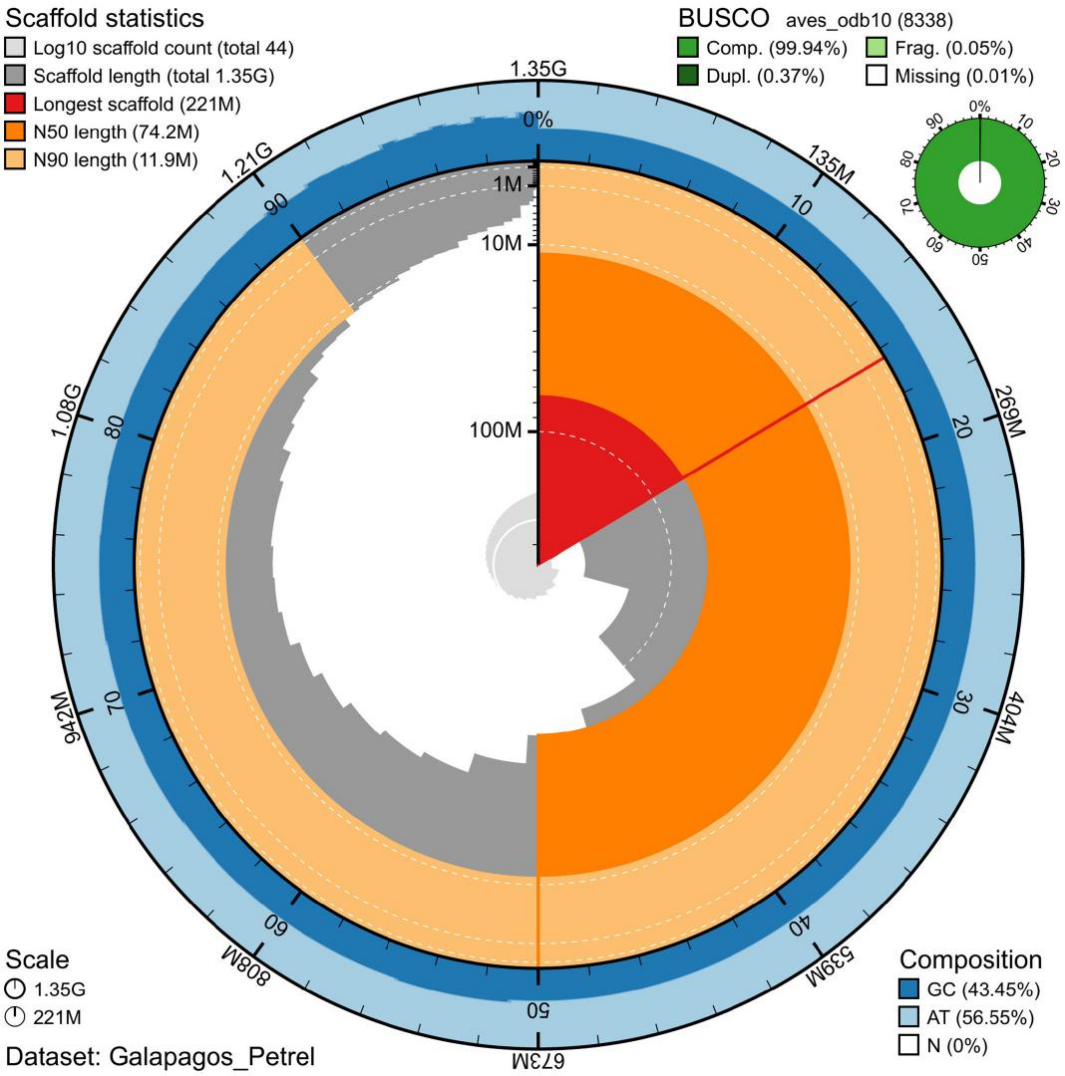
Blobtoolkit snail plot summarizing assembly metrics for the Galápagos Petrel (*P. phaeopygia*) genome. The longest scaffold is indicated in red; N50 and N90 scaffold lengths are marked in dark orange and light orange, respectively. GC content is shown in dark blue and AT content in light blue. BUSCO completeness is summarized in the inset table.

To place the Galápagos Petrel genome in a phylogenetic context, we constructed a BUSCO-based phylogeny including our assembly, 16 genomes from order Procellariiformes, and one outgroup species (Fig. 3). We used the published genomes from the Cory's Shearwater (*C. borealis*, GCA_964195595.2), Great Shearwater (*A. gravis*, GCA_045784155.1), Balearic Shearwater (*Puffinus mauretanicus,* GCA_023333565.1), Yelkouan Shearwater (*Puffinus yelkouan,* GCA_045787345.1), Common Diving Petrel (*P. urinatrix*, GCA_013400755.1), Northern Fulmar (*Fulmarus glacialis*, GCA_000690835.1), Wedge-rumped Storm Petrel (*Oceanodroma tethys*, GCA_013397025.1), Leach's Storm Petrel (*Oceanodroma leucorhoa*, GCA_030449065.1), Wilson's Storm Petrel (*Oceanites oceanicus,* GCA_013396615.1), White-bellied Storm Petrel (*Fregetta grallaria*, GCA_013399335.1), Black-footed Albatross (*Phoebastria nigripes*, GCA_035582835.1), Laysan Albatross (*Phoebastria immutabilis*, GCA_035582775.1), Short-tailed Albatross (*Phoebastria albatrus*, GCA_035582865.1), Waved Albatross (*Phoebastria irrorata*, GCA_035582855.1), Snowy Albatross (*Diomedea exulans*, GCA_035582875.1), and Black-browed Albatross (*Thalassarche melanophris*, GCA_045784095.1), as the closest relatives to the Galápagos Petrel with published, high-quality genomes. The African Penguin (*Spheniscus demersus*, GCA_050947425.1), a closely related non-Procellariiform species, served as our outgroup.

Genome assemblies were downloaded from NCBI and assessed for completeness using Compleasm v2.06 (Huang and Li, 2023) with the aves_odb10 lineage dataset. The odb10 version was selected over the newly released odb12 lineage dataset as it includes a larger set of conserved single-copy orthologs, providing greater phylogenetic resolution. BUSCO assessments were run using a custom script (see Code Availability).

Species BUSCO files created by Compleasm were compared to ensure that only single-copy orthologs present across all species were retained for alignment and tree construction.

Each ortholog group was aligned across species using MAFFT v7.525 (Katoh and Standley 2013), and alignments were trimmed where necessary to remove poorly aligned regions using trimAl v1.5.rev0 (Capella-Gutiérrez et al. 2009). These alignments were then concatenated into a single supermatrix representing shared BUSCO genes across taxa. Phylogenetic inference was conducted using IQ-TREE v2.4.0 (Minh et al. 2020) under a maximum-likelihood framework. Model selection and ultrafast bootstrap analysis were performed within IQ-TREE to assess node support and ensure robust phylogenetic inference.

## Results

### Basecalling, filtering, and correction

The two basecalled runs produced 4,101,873 reads totaling 49.1 Gbp. Trimmed data have 4,103,512 reads, slightly more due to reads split by Porechop_ABI, and 48.69 Gbp with an average coverage for a 1.35 Gbp genome of 36.07×, mean read length 11,866, and N50 22,496. Almost all reads have average quality Q10 or greater, and those with Q20 or greater have over 23× of coverage (Supplementary Table 1, Supplementary Fig. 3).

Dorado corrected reads reduced the read count to 2,403,479, though the number of bases retained, at 42.66 Gb, is 87.6% of the precorrected bases, representing 31.6× genome coverage. N50 improved slightly to 24,321. Quality scores are not available since Dorado correct output is in fasta file format, however, the method is intended to raise the read mean quality to a minimum of Q10. Though relative to Hifiasm –ont, “on a few limited datasets herro [i.e. Dorado correct] gives slightly better QV (by ∼1 in the Phred scale)” (https://github.com/chhylp123/hifiasm/issues/742).

Hifiasm corrected reads increased Q20 mean read bases by 25% (39.04 Gbp after, 31.16 Gbp before) and more than 3.5 times more Q25 mean read bases (18.68 Gbp after, 5.1 Gbp before).

### Genome size estimate

Genome size estimates of the 21, 25, and 27 length kmer histograms ranged from 1.238 to 1.239 Gb, heterozygosity 0.47% to 0.54%, and repeat length of 8.95% to 10.57% of the size, a likely underestimate of the repeats, as results following indicate. Unique length estimates ranged from 1.108 to 1.128 Gb, with the final assembly's nonrepeat length in the middle of this range.

### Genome assembly

The Hifiasm v0.24 –ont contig assembly scored best in six metrics used to assess assembly quality (Table 1). Hifiasm v0.24 uses its own error correction (Cheng et al. 2025), whereas Hifiasm v0.20 used dorado-corrected reads, as did Flye. The Flye v2.9.5 metrics were second in each category except the number of contigs (Supplementary Tables 2 to 4). Compleasm plus BUSCO aves_odb10 results were near full completeness for all three, with a single BUSCO missing in each of the top two assemblies (OrthoDB 48114at8782) and two missing in the other (Table 1).

These assembly results contrast with those found in the assembly of the lava gull (*Leucophaeus fuliginosus*), in which Flye was the top-performing assembler (Martin et al. 2025, unpublished manuscript). These contrasting results are notable given that both studies used the same laboratory and sequencing protocols; however, our study included two sequencing runs, resulting in approximately 60% greater coverage. Thus, the performance difference in our assemblies may reflect Hifiasm's ability to take full advantage of increased depth through its built-in error correction and graph-based haplotype resolution, both of which benefit significantly from higher coverage.

Hifiasm v0.24 was rerun after removing contaminant-identified reads, resulting in identical output, confirming FCS results finding no contamination in the assembly, and that any mitochondria had been assembled in circular sequences, which were excluded in gfa to fasta conversion. Purged_dups yielded an assembly of 1.346 Gb with 61 contigs and reduced BUSCO duplicates to 31, increased N90 to 10.8M from 8.8M, and left the largest contig, N50 and the single Missing BUSCO unchanged (Supplementary Table 5).

The RagTag scaffolded genome with *C. borealis* hap1 used as a reference contains 44 records after gap filling. Twelve records were scaffolded with two or three input contigs (eight 2, four 3 contigs) introducing 16 gaps, the rest remaining as contigs. Four of these 16 gaps were filled in three scaffolds, converting them to contigs with no gaps present. This includes the largest in the assembly, pseudo-chromosome 1, which also has both telomeres. Table 3 shows the progression from the initial contig assembly, the duplicate purged assembly, and finally the gap-filled scaffolded assembly containing 12 gaps we have named bPteroPhaeo_1.0. (Table 3).

**Table 3.** Improvements through the three stages of the assembly pipeline.

Assembly	Num. records	Length	Longest record	N50/L50	N90/L90
Contig	109	1.408G	207.3M	36.27M/8	8.79M/39
Dup. purged	61	1.346G	207.3M	36.27M/8	10.8M/33
Final scaffolded	44	1.346G	221.23M	74.2M/5	10.8M/30

Although we recognize that reference-guided scaffolding can be sensitive to interspecific structural variation, we mitigated this risk by retaining only high-confidence joins (12 total in 9 scaffolds) that were supported by consistent alignment evidence (Supplementary Table 6). The limited number of scaffold joins and their internal consistency suggest that large-scale structural differences between the Galápagos Petrel and Cory's Shearwater genomes are minimal at this resolution. Nevertheless, as portable sequencing options become more accessible, future incorporation of long-range chromatin contact data (e.g. Hi-C or Pore-C) could provide an independent validation of large-scale genome organization and further refine the assembly.

**Fig. 4.**
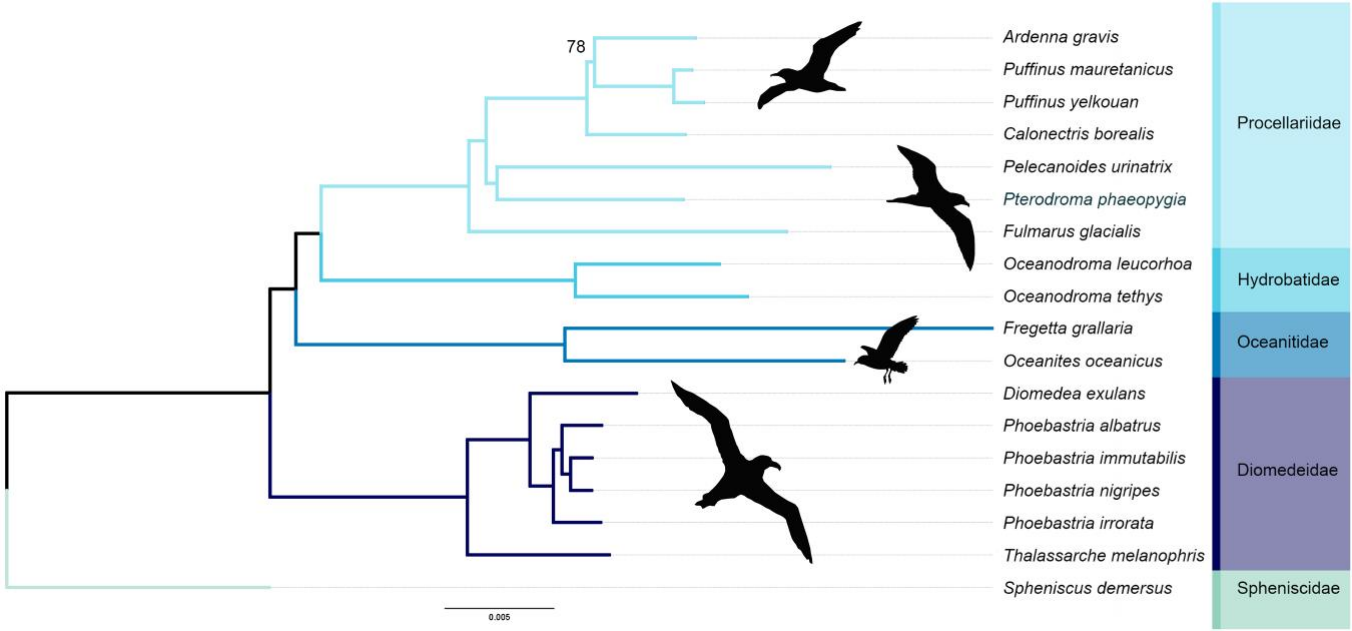
BUSCO-based phylogeny of the Galápagos Petrel (*P. phaeopygia*) and related procellariiformes. A maximum-likelihood phylogeny was constructed using 6,357 single-copy BUSCO orthologs shared among 18 Procellariiform species using IQ-TREE. All nodes received 100% bootstrap support except for the Ardenna-Puffinus node (78%).

The final genome assembly is approximately 1.35 Gb in size, consistent with, though on upper end of, medium-sized seabird genomes (e.g. *C. borealis* at 1.37 Gb, 1.2 Gb *P. urinatrix* [NCBI GCA_013400755.1]). A scaffold N50 of 74.2 Mb and L50 of 5 indicate that half of the genome assembly is contained within just five scaffolds (Table 3, Supplementary Table 7), consistent with the largest macrochromosomes typically observed in avian karyotypes (Kretschmer et al. 2018; Kiazim et al. 2021). In homology with *C. borealis,* we identified 41 records as pseudo-chromosomes, of which 23 are telomere-to-telomere (T2T; gap-free sequenced spanning both chromosomal ends) and 16 have one telomere, including the sex chromosomes, W and Z (Supplementary Table 8). Chromosome assembly records can be found in Supplementary Section 3.1. All but one of the 8,338 BUSCO aves_odb10 orthologs are found. These values indicate a high-quality genome assembly (Fig. 4, Supplementary Table 8).

Comparisons of *P. phaeopygia* and *C. borealis* assemblies based on BUSCO orthologs from the aves_odb10 dataset identified only one missing BUSCO for this assembly (*bPteroPhaeo_1.0*), while the *C. borealis* assembly is missing that same ortholog plus one additional gene (Supplementary Fig. 4). As a result, nearly all of the 8,338 aves_odb10 BUSCOs are represented in the comparison, underscoring the high genomic completeness and structural similarity between the two assemblies. Several microchromosomes lacked annotated BUSCOs in both assemblies. For these regions, we used RagTag alignment results to identify sequence agreement and visualized them as black ribbons in the Circos plot (Supplementary Fig. 4).

### Repeat analysis

RepeatMasker analysis using the Dfam28 curated Aves dataset combined with de novo repeats from RepeatModeler reports 17.43% of the total assembly sequence as repeats at 234.7 Mb (Supplementary Fig. 5, Supplementary Table 9). The remaining 1.112 Gb total nonrepeat sequence is 3 Mb from the kmer 21 GenomeScope2 estimate, less than a 1% difference; whereas the repeat content estimates are each at least 100 Mb lower than those identified by RepeatMasker (Supplementary Figs. 5 and 6, Supplementary Table 9).

### Genome annotation

BRAKER3 ab initio annotation, after refinement requiring start and stop codons and excluding embedded genes, presented candidate models for 29,316 genes and 31,448 mRNAs (genes and isoforms) (Supplementary Table 10). Functional analysis, which requires identified protein domains or homology with existing genes, retained models for 27,344 genes and 29,343 mRNAs. Compleasm running in protein mode found 7,811, 93.68%, of the aves BUSCOs in the protein AA sequences (Table 4). OMArk identified input as belonging to Neognathae and reported results from the Hierarchical Orthologous Groups associated with this clade (Supplementary Fig. 7).

**Table 4.** Basic gene model statistics.

Annotation type	Value
Number of genes	27,244
Genome percentage	15.23%
Number of mRNA	29,343
mRNA mean length	8,143 bp
Mean exons per mRNA	6.56
Mean mRNA exon len.	174.46
Single exon genes	5,895
Named genes	20,130
Named mRNA	22,188
BUSCO aves_odb10 lineage	93.68%/7,811

### Mitochondrial assembly and annotation

The *P. phaeopygia* consensus mitochondrial sequence derived from 324 mitochondrial HiFi reads is 17,315 bp with 22 tRNAs, 13 protein coding genes, and two rRNAs, as typical in birds and most vertebrates (Supplementary Table 11). The control region of 1,748 bp has a 138 bp consensus repeat motif repeated 3.9 times spanning 538 bp. This small number of mitochondrial records is likely due to the use of the ultra-long method with selected molecules for sequencing typically longer than vertebrate mitochondria. An annotated representation of mitochondrial gene order and structure is provided in Supplementary Fig. 8.

### Genome comparison

The Galápagos Petrel genome size was estimated at 1.35 Gb, consistent with the range observed across Procellariiforms (1.2 to 1.4 Gb). The assembly achieved 36× coverage and contained only 44 scaffolds, representing a highly contiguous assembly compared to previously published genomes, which range from 354 to over 51,000 scaffolds. The longest scaffold in the Galápagos Petrel assembly reached 221.2 Mb, closely matching the chromosome-scale assembly of *C. borealis* (221.1 Mb). Other metrics further support the high quality of the present assembly, including the N50 values and the high BUSCO completeness score in relation to other assemblies (Table 2).

### BUSCO phylogeny

Six thousand three hundred and fifty-seven of the 8,338 BUSCO orthologs were shared by all 18 species and thus were included in the phylogenetic analysis. All nodes in both the Procellariiformes BUSCO phylogeny were supported with bootstrap values of 100, except the *Ardenna-Puffinus* node, which had a value of 78. The constructed BUSCO phylogeny places the Galápagos Petrel (*P. phaeopygia*) as a sister group with *P. urinatrix*. This placement agrees with a previous phylogenetic analysis based on genome-wide markers, which used a similar outgroup and recovered near-identical relationships (Levy et al. 2023).

## Discussion

The successful generation of a high-quality reference genome for the critically endangered Galápagos Petrel (*P. phaeopygia*) represents a significant contribution to seabird genomic research. Our assembly, generated from ultra-long ONT reads and scaffolded using a closely related species, demonstrates exceptional quality with a scaffold N50 of 74.2 Mb and only 12 gap regions. It achieves near-complete gene content (i.e. BUSCO aves_odb10 score of 99.94% and only a single missing ortholog) and chromosomal-level resolution (i.e. 41 pseudo-chromosomes: 39 autosomal, W and Z, including 23 telomere-to-telomere). These metrics are consistent with benchmarks for high-quality reference genomes (Huang and Li 2023; Park et al. 2023), supporting its utility in downstream applications.

Our BUSCO phylogeny further supports the robustness of the assembly, recovering well-resolved relationships across Procellariiformes and placing the Galápagos Petrel as sister to *P. urinatrix* (Common Diving Petrel), a topology consistent with previous analyses.

Comparisons with other Procellariiform genomes further highlight the exceptional contiguity of this assembly, which contains fewer scaffolds than closely related genomes and achieves high completeness despite relying solely on ONT long reads.

Importantly, this study demonstrates that ONT-only workflows are now capable of producing very high quality, highly contiguous genome assemblies, challenging the perception that such assemblies require PacBio HiFi data or additional scaffolding technologies. Recent advances in ONT basecalling algorithms have substantially improved per-read accuracy, approaching values historically associated with HiFi sequencing. Moreover, modern assemblers such as HiFiasm can incorporate ONT-specific error correction models that yield assemblies comparable to HiFi-based datasets. We were pleasantly surprised to find that ultra-long, high-molecular weight ONT reads alone produced a well-supported, chromosome-scale assembly with clean statistics and minimal polishing, underscoring the emergence of ONT technology as a standalone platform for genome projects—even in remote or resource-limited field settings.

This assembly is the first genome published for a species in the genus *Pterodroma* and only the second within the Procellariidae family at chromosomal resolution, filling a significant taxonomic gap. Previous genetic research on the Galápagos Petrel was limited to microsatellite markers and mitochondrial DNA (Browne et al. 1997; Friesen et al. 2006), which, while useful, provides an incomplete and potentially biased view of genome-wide diversity. The availability of a full reference genome now enables deeper analyses of population structure, genome-wide variation, repeat content, and evolutionary history, greatly expanding the scope of research questions that can be addressed.

Notably, the identification and assembly of the W and Z sex chromosomes add critical value to this genomic resource. In many birds, sex chromosomes are hotspots for differentiation, harbor loci involved in reproductive isolation, and often exhibit different evolutionary dynamics than autosomes (Hooper et al. 2019; Luohao et al. 2019). Their inclusion facilitates investigations into sex-biased dispersal (Brom et al. 2018), differential gene expression (Mank and Ellegren 2009), and the potential role of sex-linked loci in local adaptation (Nadachowska-Brzyska et al. 2019).

In addition to providing a valuable genomic resource for avian research, our findings have broad implications for conservation. The strong natal philopatry exhibited by *P. phaeopygia* (Cruz and Cruz 1987) suggests that populations may be highly structured, potentially increasing their vulnerability to inbreeding and local extinction. With this genome, we will be able to genotype individuals across populations to assess gene flow, inbreeding levels, and fine-scale population differentiation, essential for defining conservation units and informing future management strategies.

Finally, the success of this project was made possible, in part, by sequencing DNA locally at the Galapagos Science Center (GSC). This approach ensured compliance with strict regulations governing the import and export of biological material, one of the limitations we faced in our sequencing pipeline. In addition, the use of portable ONT devices demonstrates the feasibility of conducting high-throughput sequencing in remote, resource-limited environments, while also contributing to the growing genomic research capacity in isolated regions such as the Galápagos Islands.

## Code availability

Custom scripts are in https://github.com/jaimechaves76/GalapaGenomes-Galapagos-Petrel-G3 github repository as well as the https://github.com/calacademy-research/assembly_etc github repository under the scripts folder. Files seq_summary_qscore_lens.sh for read statistics and asmstats.pl for assembly statistics are located there, as are script dependencies and basic_gff_stats.sh. Several scripts also use bioawk_cas from https://github.com/calacademy-research/bioawk.CAS, an enhanced version of Heng Li's bioawk (https://github.com/lh3/bioawk).

## Supplementary Material

jkag004_Supplementary_Data

## Data Availability

Raw sequencing data is available on NCBI under BioProject PRJNA1275046 and BioSample SAMN49010279: GAPE_003. The genome assembly is deposited under the same BioProject on NCBI. All NCBI submissions will be made public at the time of publication. GFF files are available at GSA FigShare: https://doi.org/10.25387/g3.29648078. Supplemental material available at G3 online.
