# Supplementary material for: First whole-genome assembly of the Galápagos Petrel (Pterodroma phaeopygia) using Oxford Nanopore sequencing to advance conservation genomics in a critically endangered seabird
Source: G3 (Bethesda). 2026 Jan 27;16(3):jkag004. doi: 10.1093/g3journal/jkag004 (PMC12958810; doi:10.1093/g3journal/jkag004)
Supplement: jkag004_Supplementary_Data [file jkag004_supplementary_data.docx]

## **Supplementary Material**

**First Whole-Genome Assembly of the Galápagos Petrel (*Pterodroma phaeopygia*) Using Oxford Nanopore Sequencing to Advance Conservation Genomics in a Critically Endangered Seabird**

Isabella R. Sessi, James B. Henderson, Jessica A. Martin, Alice Skehel,

Gabriela Pozo, Jonathan A. Guillén Alcides, Vera de Ferran, John P. Dumbacher,

Jaime A. Chaves

**Section 1: Sequencing and Quality Assessment**

**1.1 Sequencing Runs and GenomeScope2**

The genome was sequenced in two independent runs. The first run from July 2024 generated ~15x coverage, however GenomeScope2 analysis using kmer length 21 showed the first peak, representing heterozygous kmers, at ~7X coverage, overlapping with error kmers and calculated an inaccurate genome haploid length of 594 Mbp and heterozygosity of 20.1%. Clearly an error for such a likely low heterozygosity avian species. A second sequencing run was conducted in January 2025 to improve depth and assembly quality and to resolve the kmer spectrum analysis with additional genome coverage. We checked the second run to see if its slightly larger ~18x standalone coverage yielded an improvement, and not surprisingly it had a similar GenomeScope2 error kmer peak in lieu of a heterozygous peak reporting haploid size 513Mb, heterozygosity 21.7%.

| 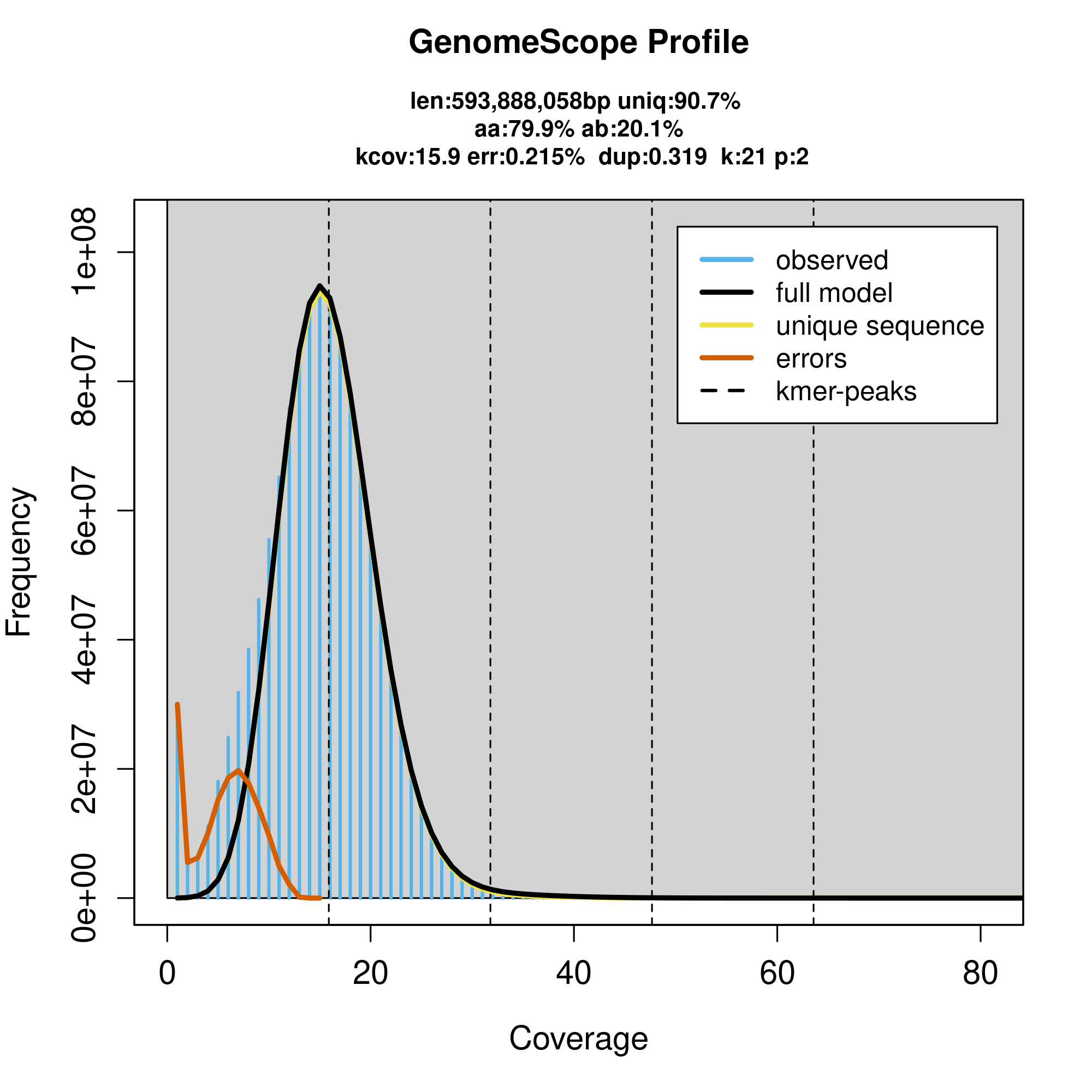 | 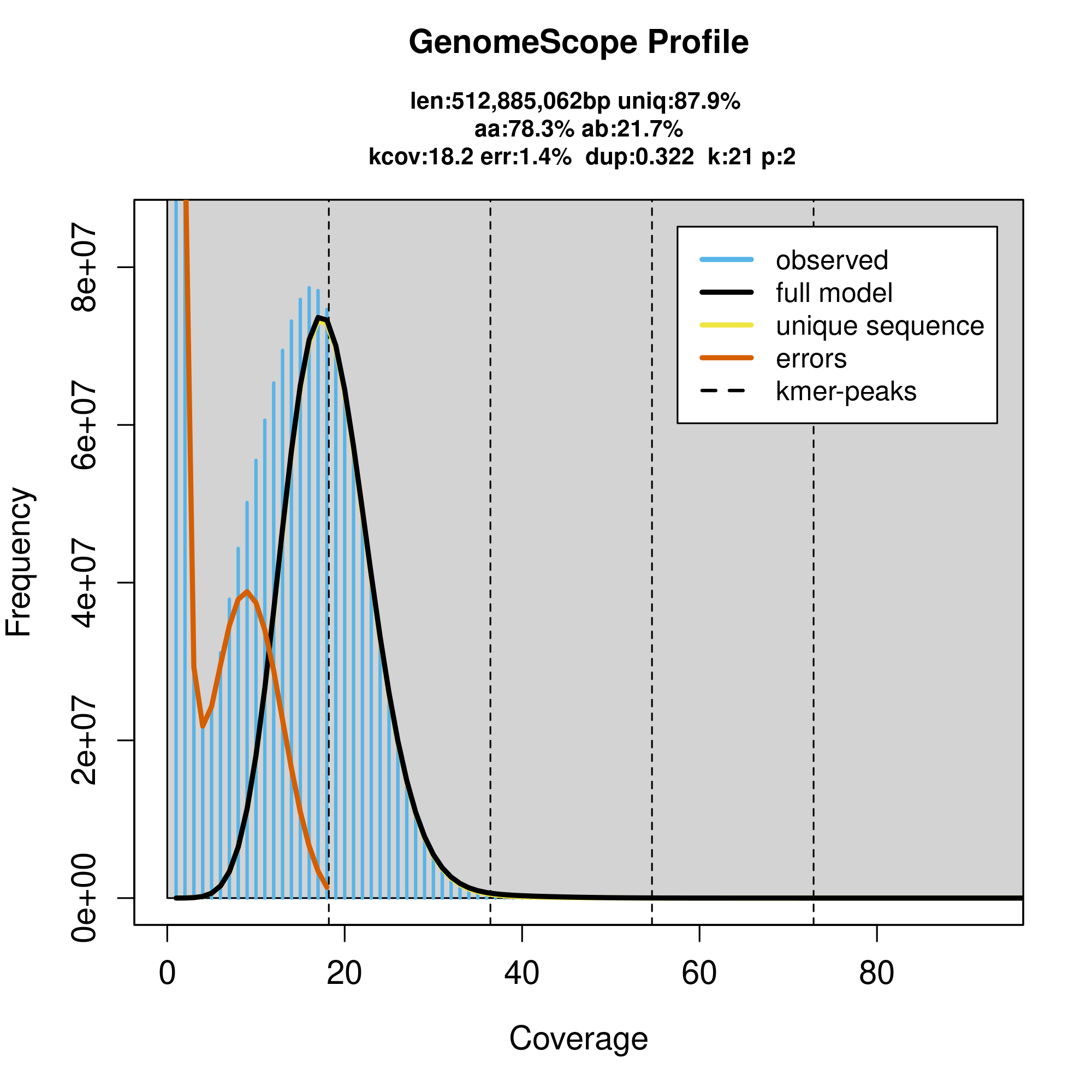 |
| --- | --- |

**Figure S1**. GenomeScope2 profile plots, kmer 21. Left, sequence run 1. Right, sequence run 2.

Both runs were combined, after trimming, for ~33x coverage and kmer 21 GenomeScope2 analysis reported 1.24 Gbp haploid size and 0.533% heterozygosity. These were much closer to the expected values for a bird with few mating pairs. Not uncommon for kmer spectrum analysis, repeat content was underestimated by the GenomeScope2 model and the 1.35 Gbp assembled genome was reported as 8% smaller by GenomeScope2, which is not an uncommon underestimate for this kmer based model.


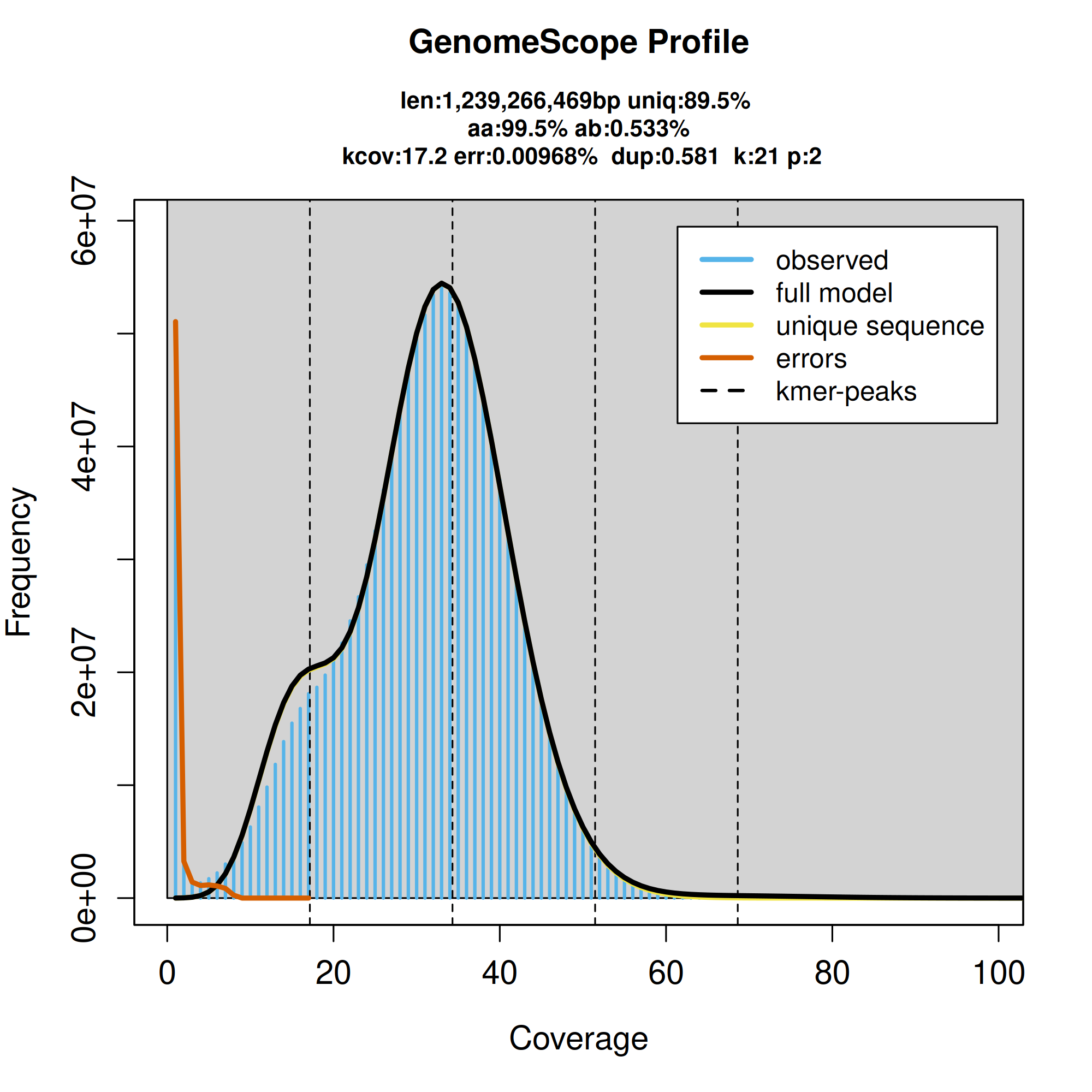


**Figure S2**. GenomeScope2 profile plot, kmer 21, of the two combined sequence runs.

**1.2 Read Statistics and Quality Filtering**

**Read Statistics**

**Table S1**. Porechop_ABI trimmed read statistics. Coverage based on 1.35G genome.

reads 4,103,512 bases 48,692,165,492 36.07X

shortest 2 longest 1,412,646

mean 11,865.97 median 6,665.00

N50 22,496 L50 644,804 N40 27,770 L40 449,807

N30 34,151 L30 291,371 N20 42,746 L20 163,380

N10 57,290 L10 63,570

Q10+ 4,096,352 48.67G 99.95% 36.05X

Q12+ 3,930,756 46.77G 96.06% 34.65X

Q15+ 3,594,223 43.00G 88.30% 31.85X

Q20+ 2,466,728 31.16G 63.99% 23.08X

Q25+ 458,408 5.10G 10.48% 3.78X

Q30+ 16,700 48.3M 0.10% 0.04X

1+ 4,103,512 48.69G 100.00% 36.07X

1000+ 4,007,242 48.61G 99.83% 36.01X

5000+ 2,397,264 44.34G 91.06% 32.85X

10000+ 1,561,752 38.31G 78.68% 28.38X

15000+ 1,083,196 32.42G 66.58% 24.01X

30000+ 386,672 17.65G 36.26% 13.08X

40000+ 196,649 11.11G 22.82% 8.23X

50000+ 100,919 6.86G 14.09% 5.08X

75000+ 23,363 2.28G 4.68% 1.69X

100000+ 7,077 900.0M 1.85% 0.67X

| 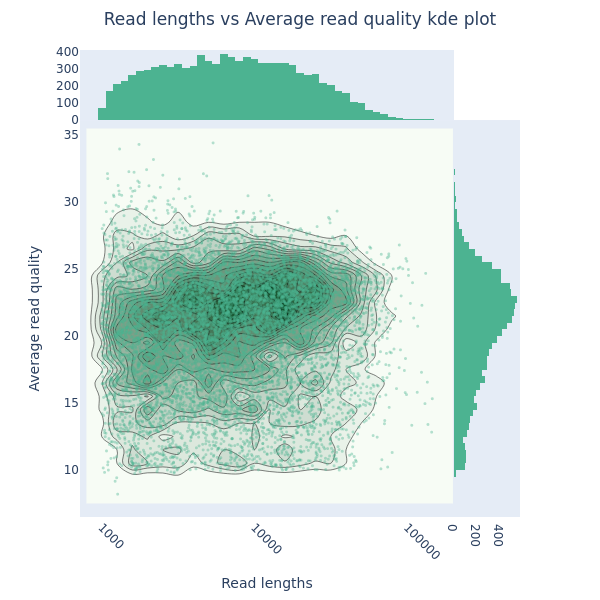 | 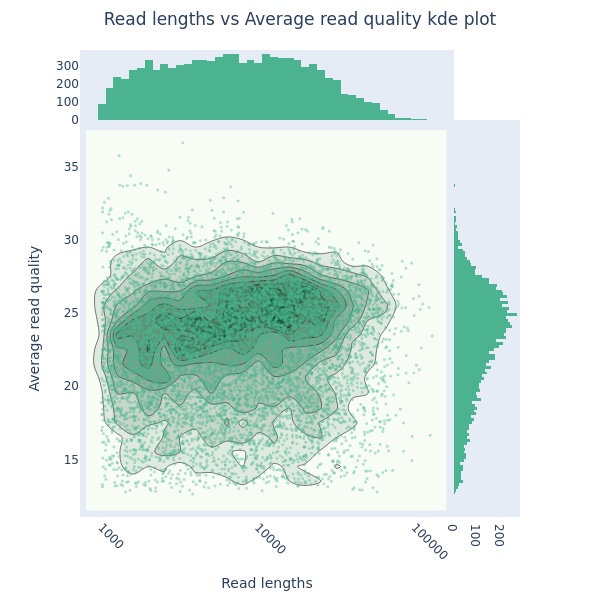 |
| --- | --- |

**Figure S3**. Nanoplot KDE plots. Read log lengths vs. Average read quality. Min 1K, Max 150K lengths.

(a) trimmed reads, y-axis numbers start at 10, (b) Hifiasm corrected reads, y-axis numbers start at 15.

**Section 2: Basecalling, Correction, and Assembly**

**2.1 Computational Environment**

**DELL laptop specs**

The Dell laptop used for sequencing was equipped with:

- 13th Gen Intel(R) Core(TM) i9-13950HX 2.20 GHz, x64-based processor
- 128 GB RAM
- 64-bit operating system, Windows 10.

This laptop was not equipped with a GPU for live basecalling. Basecalling was originally attempted on the DELL laptop, which caused it to crash. We subsequently decided to complete sequencing and basecalling separately.

**2.2 Contig-level and Pre-scaffolding Assemblies**

**Contig-level assembly statistics**

Hifiasm v0.24 using basecalled then trimmed, but otherwise uncorrected reads, was chosen as the best assembly. The statistics for the three assemblies are in the following tables in assessed order.

**Table S2**. Assembly and aves_odb10 statistics for Hifiasm –ont on uncorrected reads.

Hifiasm v0.24: hifiasm --write-ec --telo-m CCCTAA -t 32 --ont trimmed_reads.fastq

Number of contigs 109

Total size of contigs 1407677265 1.408G

Longest contig 207298667 207.299M

Shortest contig 4694

Number of contigs > 1M nt 76 69.7%

Number of contigs > 10M nt 35 32.1%

auN50 69017952 69.184M

N50 contig length 36264505 L50 contig count 8 36.265M

N90 contig length 8787604 L90 contig count 39 8.788M

C:8334[S:8276,D:58],F:3,M:1,n:8338

C:99.95%[S:99.26%,D:0.70%],F:0.04%,M:0.01%,n:8338

**Table S3**. Assembly and aves_odb10 statistics for Flye on corrected reads.

Flye v2.9.5: flye --scaffold -t 32 --nano-corr corrected_reads.fasta

Number of scaffolds 1038

Total size of scaffolds 1309516820 1.31G

Longest scaffold 104335507 104.336M

Shortest scaffold 513

Number of scaffolds > 1M nt 126 12.1%

Number of scaffolds > 10M nt 35 3.4%

auN50 32811490 32.811M

N50 scaffold length 20929519 L50 scaffold count 17 20.93M

N90 scaffold length 2138548 L90 scaffold count 93 2.139M

C:8333[S:8275,D:58],F:4,M:1,n:8338

C:99.94%[S:99.24%,D:0.70%],F:0.05%,M:0.01%,n:8338

**Table S4**. Assembly and aves_odb10 statistics for Hifiasm on corrected reads.

Hifiasm v0.20.0: hifiasm --telo-m CCCTAA -t 32 corrected_reads.fasta

Number of contigs 918

Total size of contigs 1445777242 1.446G

Longest contig 26216668 26.217M

Shortest contig 6568

Number of contigs > 1M nt 251 27.3%

Number of contigs > 10M nt 33 3.6%

auN50 8194306 8.194M

N50 contig length 7288658 L50 contig count 64 7.289M

N90 contig length 1164726 L90 contig count 234 1.165M

C:8329[S:7706,D:623],F:7,M:2,n:8338

C:99.89%[S:92.42%,D:7.47%],F:0.08%,M:0.02%,n:8338

**Table S5**. Assembly and aves_odb10 statistics for purge_dups of hifiasm v0.24 –ont on uncorrected reads. These statistics reveal further improvement in the length statistics and minor BUSCO score reduction in number of fragments (3 to 4).

Purge_dups.sh v1.2.5 hifiasm_–ont_trimmed_reads.fasta

Number of contigs 61

Total size of contigs 1346316474 1.346G

Longest contig 207298667 207.299M

Shortest contig 4694

Number of contigs > 1M nt 58 95.1%

Number of contigs > 10M nt 33 54.1%

auN50 71811103 71.811M

N50 contig length 36264505 L50 contig count 8 36.265M

N90 contig length 10797075 L90 contig count 33 10.797M

C:8333[S:8302,D:31],F:4,M:1,n:8338

C:99.94%[S:99.57%,D:0.37%],F:0.05%,M:0.01%,n:8338

**2.3 Gap Validation Prior to Final Scaffolding**

To evaluate the reliability of our reference-based scaffolding approach, we examined gene collinearity around assembly gaps using BUSCO orthologs in comparison with the orthologs of an assembly from a closely related species, *Ardenna gravis* (Great Shearwater), and assessed whether the two flanking genes occur adjacently on a single scaffold in the reference.

For each scaffold gap in the Galápagos Petrel assembly, we identified the nearest BUSCO genes flanking the gap (the closest complete BUSCO upstream and downstream). Across the Galápagos Petrel assembly, 12 scaffolds contained gaps; two of these lacked flanking BUSCOs. Overall, 7 of 10 evaluable gaps were supported by conserved BUSCO adjacency in *A. gravis*, providing additional confidence in the accuracy of reference-guided scaffold joins (Table S6).

**Table S6**. Gaps Supported by *Ardenna gravis* Assembly

| BUSCO | Rec | # | Tot | Start | End | Type | BUSCO | Rec | # | Tot | Start | End | Type |
| --- | --- | --- | --- | --- | --- | --- | --- | --- | --- | --- | --- | --- | --- |
| 8891at8782 | Pphae2 | 340 | 769 | 72532678 | 72736239 | Complete | 8891at8782 | JBJGHL010013150.1 | 2 | 25 | 580539 | 781936 | Duplicate |
| Scaffold Gap | Pphae2 | . | . | 72912800 | 72912899 | +176561 | -380652 | GAP SUPPORTED |  |  |  |  |  |
| 39325at8782 | Pphae2 | 341 | 769 | 73293353 | 73298091 | Complete | 39325at8782 | JBJGHL010013150.1 | 1 | 25 | 13339 | 18068 | Duplicate |
|  |  |  |  |  |  |  |  |  |  |  |  |  |  |
| 41695at8782 | Pphae2 | 346 | 769 | 75811261 | 75828804 | Complete | 41695at8782 | JBJGHL010029813.1 | 5 | 6 | 2376848 | 2394314 | Complete |
| Scaffold Gap | Pphae2 | . | . | 76825618 | 76825717 | +996814 | -251659 | GAP SUPPORTED |  |  |  |  |  |
| 5029at8782 | Pphae2 | 347 | 769 | 77077178 | 77193823 | Complete | 5029at8782 | JBJGHL010029813.1 | 6 | 6 | 3614601 | 3729389 | Complete |
|  |  |  |  |  |  |  |  |  |  |  |  |  |  |
| 35789at8782 | Pphae3 | 68 | 736 | 11958611 | 11959953 | Complete | 35789at8782 | JBJGHL010029829.1 | 15 | 236 | 3506478 | 3507821 | Complete |
| Scaffold Gap | Pphae3 | . | . | 12146489 | 12146588 | +186536 | -290443 | GAP SUPPORTED |  |  |  |  |  |
| 36224at8782 | Pphae3 | 69 | 736 | 12436833 | 12442919 | Complete | 36224at8782 | JBJGHL010029829.1 | 16 | 236 | 3984027 | 3986992 | Complete |
|  |  |  |  |  |  |  |  |  |  |  |  |  |  |
| 18449at8782 | Pphae8 | 124 | 316 | 14124474 | 14130181 | Complete | 18449at8782 | JBJGHL010001619.1 | 27 | 69 | 3582182 | 3587918 | Complete |
| Scaffold Gap | Pphae8 | . | . | 14307145 | 14307244 | +176964 | -221614 | GAP SUPPORTED |  |  |  |  |  |
| 30463at8782 | Pphae8 | 125 | 316 | 14528660 | 14542228 | Complete | 30463at8782 | JBJGHL010001619.1 | 26 | 69 | 3177092 | 3190573 | Complete |
|  |  |  |  |  |  |  |  |  |  |  |  |  |  |
| 5478at8782 | Pphae13 | 36 | 181 | 5635351 | 5665981 | Complete | 5478at8782 | JBJGHL010000288.1 | 39 | 73 | 6226780 | 6257459 | Complete |
| Scaffold Gap | Pphae13 | . | . | 6609116 | 6609215 | +943135 | -1264983 | GAP SUPPORTED |  |  |  |  |  |
| 33470at8782 | Pphae13 | 37 | 181 | 7874000 | 7882245 | Complete | 33470at8782 | JBJGHL010000288.1 | 38 | 73 | 3990111 | 4005467 | Complete |
|  |  |  |  |  |  |  |  |  |  |  |  |  |  |
| 29032at8782 | Pphae20 | 79 | 171 | 5947101 | 5967699 | Complete | 29032at8782 | JBJGHL010000202.1 | 44 | 66 | 4183634 | 4204222 | Complete |
| Scaffold Gap | Pphae20 | . | . | 6307919 | 6308018 | +340220 | -488246 | GAP SUPPORTED |  |  |  |  |  |
| 29219at8782 | Pphae20 | 80 | 171 | 6796066 | 6859137 | Complete | 29219at8782 | JBJGHL010000202.1 | 45 | 66 | 5026487 | 5090123 | Complete |
|  |  |  |  |  |  |  |  |  |  |  |  |  |  |
| 43758at8782 | Pphae23 | 105 | 138 | 7520321 | 7528556 | Complete | 43758at8782 | JBJGHL010000037.1 | 35 | 37 | 2322860 | 2330249 | Complete |
| Scaffold Gap | Pphae23 | . | . | 7766046 | 7766145 | +237490 | -195883 | GAP SUPPORTED |  |  |  |  |  |
| 11845at8782 | Pphae23 | 106 | 138 | 7961830 | 7997808 | Complete | 11845at8782 | JBJGHL010000037.1 | 36 | 37 | 2347311 | 2383205 | Complete |

**Section 3: Chromosome-Level Assembly Details**

**3.1 Final Chromosome-Scale Assembly**

**Table S7**. (a) Assembly statistics of chromosome-level scaffolds. (b) Compleasm and BUSCO aves_odb10 results.

1. Information for assembly **bPteroPhaeo_1.0.fasta**

Number of scaffolds 44

Total size of scaffolds 1346342813

Longest scaffold 221226871

Shortest scaffold 897705

Number of scaffolds > 1K nt 44 100.0%

Number of scaffolds > 10K nt 44 100.0%

Number of scaffolds > 100K nt 44 100.0%

Number of scaffolds > 1M nt 43 97.7%

Number of scaffolds > 10M nt 27 61.4%

Mean scaffold size 30598700

Median scaffold size 14458064

N50 scaffold length 74196310 L50 scaffold count 5

N60 scaffold length 43645219 L60 scaffold count 8

N70 scaffold length 32354393 L70 scaffold count 11

N80 scaffold length 25694805 L80 scaffold count 16

N90 scaffold length 11894639 L90 scaffold count 24

scaffold %A 28.12 scaffold %T 28.43

scaffold %G 21.79 scaffold %C 21.66

Number of N 1200

Number of contigs 56

Number of contigs in scaffolds 21

Number of contigs not in scaffolds 35

Total size of contigs 1346341613

Longest contig 221226871

Shortest contig 82860

Number of contigs > 1K nt 56 100.0%

Number of contigs > 10K nt 56 100.0%

Number of contigs > 100K nt 55 98.2%

Number of contigs > 1M nt 54 96.4%

Number of contigs > 10M nt 30 53.6%

Mean contig size 24041815

Median contig size 11894639

N50 contig length 46848837 L50 contig count 7

N60 contig length 32354393 L60 contig count 10

N70 contig length 26044654 L70 contig count 15

N80 contig length 19720596 L80 contig count 21

N90 contig length 10797075 L90 contig count 30

(b) Compleasm and BUSCO lineage aves_odb10 n:8338

8333 Complete BUSCOs (C) 99.94%

8302 Complete and single-copy BUSCOs (S) 99.57%

31 Complete and duplicated BUSCOs (D) 0.37%

4 Fragmented BUSCOs (F) 0.05%

1 Missing BUSCOs (M) 0.01%

8338 Total BUSCO aves_odb10 group 100.00%

Missing BUSCO info:

48114at8782 glyoxalase domain-containing protein 5 <https://v10-1.orthodb.org/?query=48114at8782>

**Table S8**. Assembly records with length, record BUSCO count and telomeres

**Length BUSCO Count Telomeres**

Pphae1 221222065 B:1257 C:1254 F:1 D:1 d:2 telo: TOP 10345 bp BOTTOM 7409 bp # T2T 1

Pphae2 172840670 B:769 C:763 F:0 D:4 d:6 telo: TOP 8907 bp BOTTOM 7641 bp # T2T 2

Pphae3 130879242 B:736 C:736 F:0 D:0 d:0 telo: BOTTOM 11375 bp

Pphae4 87591918 B:456 C:445 F:0 D:4 d:11 telo: TOP 11484 bp BOTTOM 7832 bp # T2T 3

PphaeZ 74196310 B:324 C:302 F:0 D:22 d:22 telo: TOP 11972 bp

Pphae5 57428649 B:389 C:389 F:0 D:0 d:0 telo: TOP 7921 bp BOTTOM 23380 bp # T2T 4

Pphae6 46848837 B:299 C:298 F:1 D:0 d:0 telo: TOP 5060 bp

Pphae7 43645219 B:313 C:313 F:0 D:0 d:0 telo: TOP 3242 bp BOTTOM 6301 bp # T2T 5

Pphae8 40351898 B:316 C:316 F:0 D:0 d:0 telo: TOP 9178 bp BOTTOM 5349 bp # T2T 6

PphaeW 36264505 B:57 C:34 F:1 D:22 d:22 telo: TOP 7789 bp

Pphae9 32354393 B:278 C:277 F:1 D:0 d:0 telo: TOP 6254 bp BOTTOM 5232 bp # T2T 7

Pphae14 28402018 B:190 C:190 F:0 D:0 d:0 telo: TOP 8215 bp

Pphae10 27120585 B:195 C:195 F:0 D:0 d:0 telo: TOP 8275 bp BOTTOM 9351 bp # T2T 8

Pphae11 26565434 B:238 C:238 F:0 D:0 d:0 telo: BOTTOM 7741 bp

Pphae12 25710281 B:218 C:218 F:0 D:0 d:0 telo: TOP 10546 bp

Pphae13 25694805 B:181 C:181 F:0 D:0 d:0 telo: TOP 7370 bp BOTTOM 6177 bp # T2T 9

Pphae15 24198594 B:179 C:179 F:0 D:0 d:0 telo: BOTTOM 7826 bp

Pphae16 21108053 B:243 C:243 F:0 D:0 d:0 telo: TOP 6631 bp BOTTOM 5999 bp # T2T 10

Pphae17 19720596 B:186 C:186 F:0 D:0 d:0 telo: TOP 5888 bp BOTTOM 6978 bp # T2T 11

Pphae18 18769164 B:232 C:232 F:0 D:0 d:0 telo: TOP 8611 bp BOTTOM 4103 bp # T2T 12

Pphae20 15442294 B:171 C:171 F:0 D:0 d:0 telo: TOP 7115 bp BOTTOM 8070 bp # T2T 13

Pphae19 14458064 B:198 C:198 F:0 D:0 d:0 telo: BOTTOM 7809 bp

Pphae21 14112371 B:177 C:177 F:0 D:0 d:0 telo: TOP 9617 bp BOTTOM 6228 bp # T2T 14

Pphae22 11894639 B:87 C:87 F:0 D:0 d:0 telo: BOTTOM 7368 bp

Pphae23 11389550 B:138 C:138 F:0 D:0 d:0 telo: TOP 8882 bp BOTTOM 5851 bp # T2T 15

Pphae26 10908141 B:124 C:124 F:0 D:0 d:0 telo: TOP 6982 bp BOTTOM 8740 bp # T2T 16

Pphae25 10797075 B:116 C:115 F:0 D:1 d:1 telo: TOP 5893 bp BOTTOM 7737 bp # T2T 17

Pphae24 9596470 B:100 C:100 F:0 D:0 d:0 telo: BOTTOM 11333 bp

Pphae27 9114372 B:47 C:47 F:0 D:0 d:0 telo: TOP 5740 bp BOTTOM 8681 bp # T2T 18

Pphae29 8802537 B:28 C:28 F:0 D:0 d:0 telo: TOP 11654 bp BOTTOM 5169 bp # T2T 19

Pphae28 8552938 B:105 C:105 F:0 D:0 d:0

Pphae39 8418871 telo: TOP 6709 bp BOTTOM 3344 bp # T2T 20

Pphae37 7253442 telo: TOP 7003 bp

Pphae34 6499643 telo: TOP 6767 bp BOTTOM 8326 bp # T2T 21

Pphae38 6377709 telo: TOP 8575 bp BOTTOM 4423 bp # T2T 22

Pphae30 5764758 B:21 C:20 F:0 D:1 d:1 telo: BOTTOM 14806 bp

Pphae36 5712385 telo: TOP 5318 bp BOTTOM 12949 bp # T2T 23

Pphae31 5176448 telo: TOP 4396 bp

unloc2 4678367 telo: BOTTOM 6164 bp

Pphae33 2892829 B:1 C:1 F:0 D:0 d:0

unloc1 2880049

Pphae32 2448679 B:2 C:2 F:0 D:0 d:0

Pphae35 1330808 telo: BOTTOM 8357 bp

unloc3 897705

**3.2 Structural Validation and Synteny**


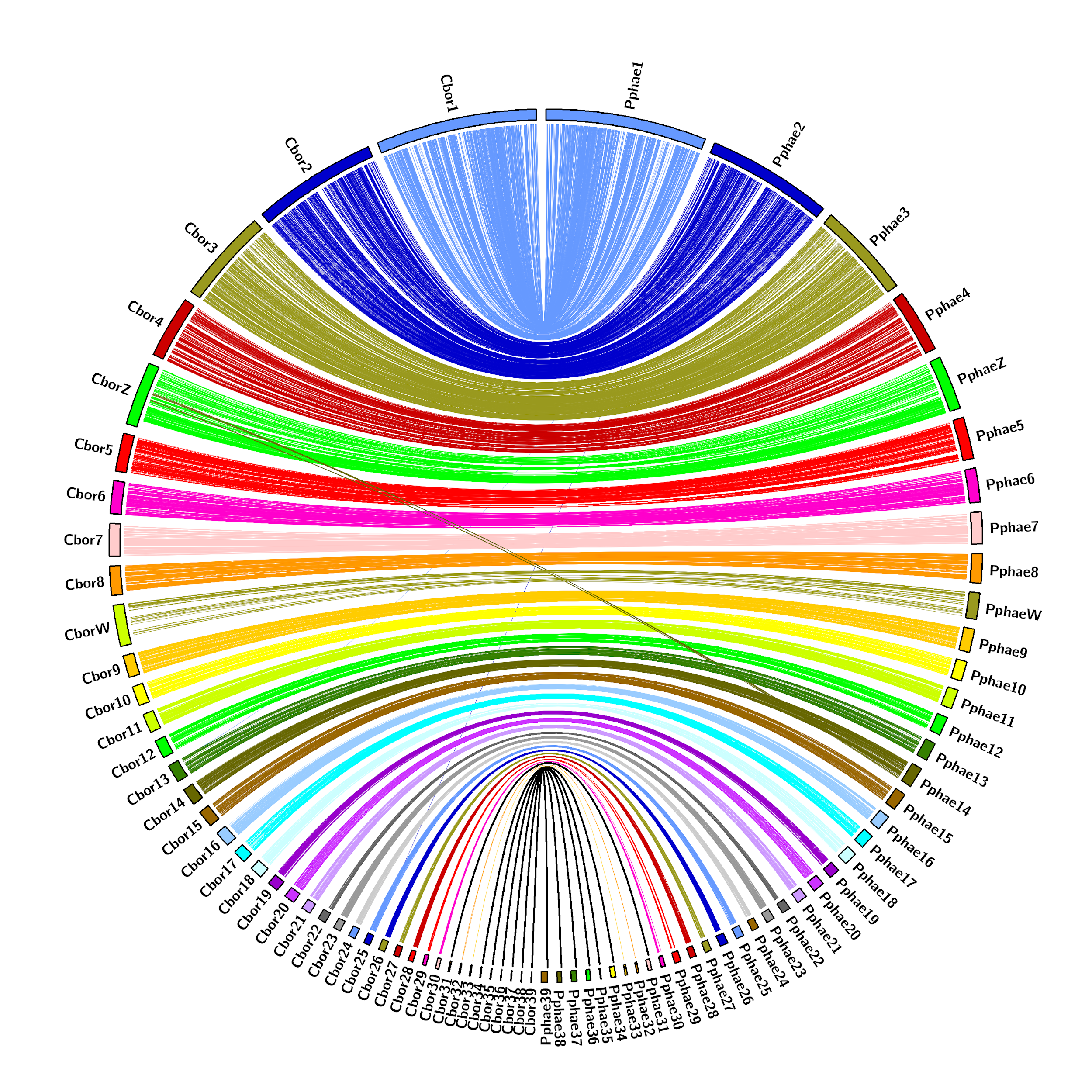


**Figure S4:** Synteny between *P. phaeopygia* and *C. borealis* using Circos plot.


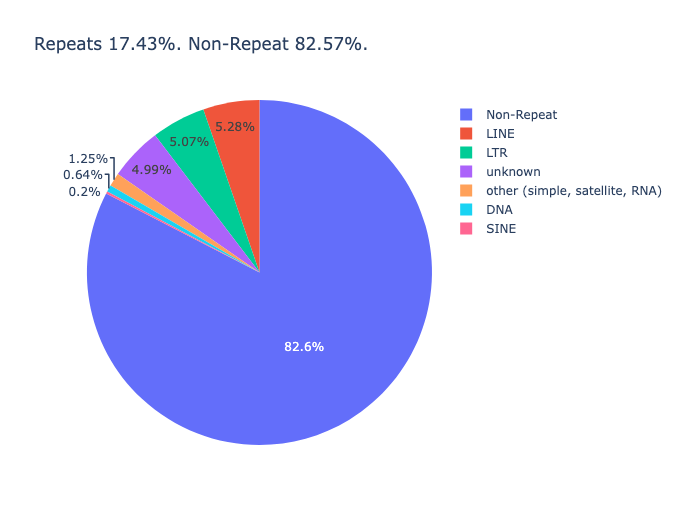


**Figure S5**. Repeat classification breakdown. Repeats 17.43%. Non-repeat 82.57%.


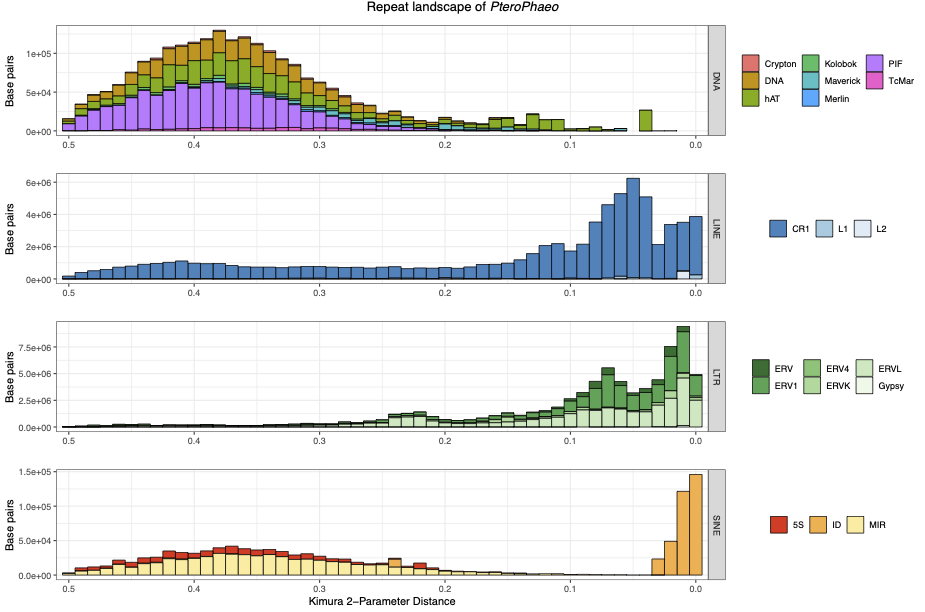


**Figure S6.** Family level classification of repeats from earlGrey.

**Figure S7:** OMArk HOGs proteome completeness analysis chart.
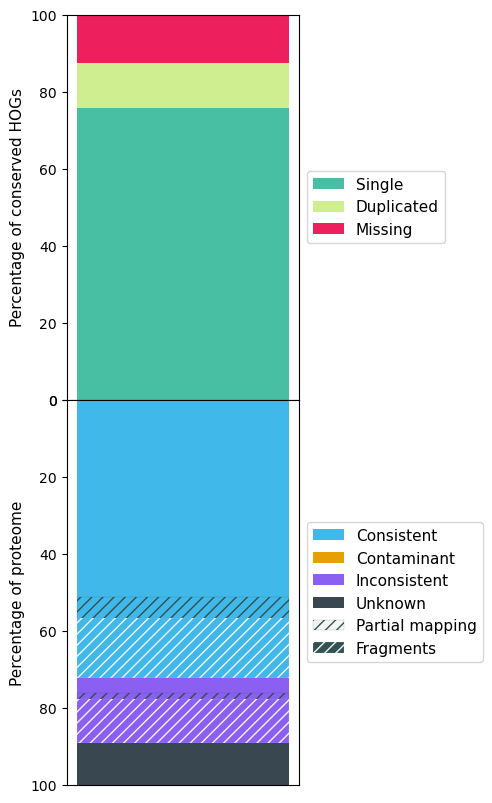


**Section 4: Repeats and Genome Composition**

**4.1 Repeat Content Statistics**

**Table S9**. Repeat and non-repeat sequence lengths per assembly records

Name Record length Non-repeat len Repeat length Repeat %

**Total 1346342813 1111633609 234709204 17.43%**

Pphae1 221226871 192720860 28506011 12.89%

Pphae2 172840670 150233467 22607203 13.08%

Pphae3 130879242 114624242 16255000 12.42%

Pphae4 87591918 77200673 10391245 11.86%

Pphae5 57437948 45990443 11447505 19.93%

Pphae6 46848837 41697425 5151412 11.00%

Pphae7 43645219 39424653 4220566 9.67%

Pphae8 40351898 34365641 5986257 14.84%

Pphae9 32354393 28867847 3486546 10.78%

Pphae10 27120585 23881126 3239459 11.94%

Pphae11 26565434 23515224 3050210 11.48%

Pphae12 25710281 22642245 3068036 11.93%

Pphae13 25694805 22891824 2802981 10.91%

Pphae14 28402018 24014325 4387693 15.45%

Pphae15 24198594 21300909 2897685 11.97%

Pphae16 21108053 18710911 2397142 11.36%

Pphae17 19720596 17191405 2529191 12.83%

Pphae18 18769164 16219438 2549726 13.58%

Pphae19 14458064 12665943 1792121 12.40%

Pphae20 15442294 13659461 1782833 11.55%

Pphae21 14112371 12546964 1565407 11.09%

Pphae22 11894639 8024522 3870117 32.54%

Pphae23 11389550 9718475 1671075 14.67%

Pphae24 9596470 8071058 1525412 15.90%

Pphae25 10797075 8070420 2726655 25.25%

Pphae26 10908141 7599749 3308392 30.33%

Pphae27 9114372 6204768 2909604 31.92%

Pphae28 8552938 6209678 2343260 27.40%

Pphae29 8802537 3667243 5135294 58.34%

Pphae30 5764758 3758314 2006444 34.81%

Pphae31 5176448 1669174 3507274 67.75%

Pphae32 2448679 1138653 1310026 53.50%

Pphae33 2892829 1506462 1386367 47.92%

Pphae34 6499643 1089456 5410187 83.24%

Pphae35 1330808 430515 900293 67.65%

Pphae36 5727713 1188121 4539592 79.26%

Pphae37 7253442 2146852 5106590 70.40%

Pphae38 6377709 878434 5499275 86.23%

Pphae39 8418871 826853 7592018 90.18%

PphaeW 36264505 19675161 16589344 45.75%

PphaeZ 74196310 63082327 11113983 14.98%

unloc1 2880049 1012386 1867663 64.85%

unloc2 4678367 1097003 3581364 76.55%

unloc3 897705 202959 694746 77.39%

**Table S10.** Select OMArk Neognathae HOGs statistics

| **OMArk statistic** | **Value** |
| --- | --- |
| Consistent | 19,707 (72.39%) |
| Inconsistent | 4,548 (16.71%) |
| Unknown | 2,969 (10.91%) |

**4.2 Mitochondrial Genome Assembly and Annotation**

**
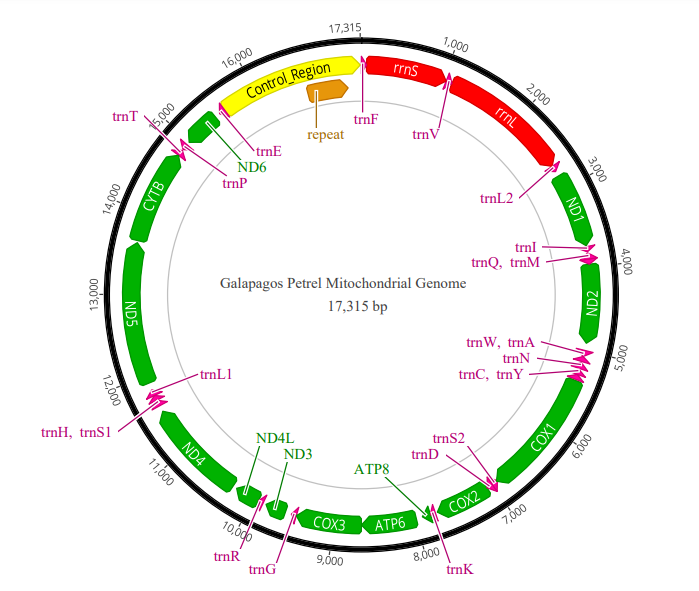
**

**Figure S8.** Annotated mitochondrial genome of the Galapagos Petrel (*Pterodroma phaeopygia*) generated using Geneious Prime.

**Table S11.** Mitochondrion annotation

# header start stop score evalue AC AA model strand

Pphae_mito 1 67 68.25 1.429E-13 GAA F Metazoa_F.cm +

Pphae_mito 67 1041 713.4 1e-184 complete rrnS 12S_rRNA.cm +

Pphae_mito 1041 1113 54.3 3.177E-10 UAC V Metazoa_V.cm +

Pphae_mito 1121 2700 1219.0 0 complete rrnL 16S_rRNA.cm +

Pphae_mito 2700 2773 43.03 7.548E-8 UAA L2 Metazoa_L2.cm +

Pphae_mito 2830 3741 424 2.43e-135 . ND1 nad1.fas +

Pphae_mito 3743 3814 54.72 8.414E-11 GAU I Metazoa_I.cm +

Pphae_mito 3823 3893 45.68 1.54E-8 UUG Q Metazoa_Q.cm -

Pphae_mito 3893 3961 58.86 1.975E-10 CAU M Metazoa_M.cm +

Pphae_mito 3962 4918 349 1.03e-108 . ND2 nad2.fas +

Pphae_mito 5001 5071 60.37 6.439E-12 UCA W Metazoa_W.cm +

Pphae_mito 5073 5141 58.45 1.586E-10 UGC A Metazoa_A.cm -

Pphae_mito 5152 5224 54.05 2.648E-10 GUU N Metazoa_N.cm -

Pphae_mito 5235 5301 45.4 9.959E-9 GCA C Metazoa_C.cm -

Pphae_mito 5302 5371 43.47 1.185E-7 GUA Y Metazoa_Y.cm -

Pphae_mito 5382 6929 790 0.0 . COX1 cox1.fas +

Pphae_mito 6924 6997 55.68 3.112E-10 UGA S2 Metazoa_S2.cm -

Pphae_mito 7000 7068 53.41 3.086E-10 GUC D Metazoa_D.cm +

Pphae_mito 7070 7729 358 6.02e-114 . COX2 cox2.fas +

Pphae_mito 7755 7824 56.03 6.002E-10 UUU K Metazoa_K.cm +

Pphae_mito 7826 7918 52.4 1.01e-09 . ATP8 atp8.fas +

Pphae_mito 7996 8664 224 6.39e-67 . ATP6 atp6.fas +

Pphae_mito 8667 9449 392 5.32e-125 . COX3 cox3.fas +

Pphae_mito 9451 9519 53.93 5.288E-10 UCC G Metazoa_G.cm +

Pphae_mito 9592 9840 117 1.08e-31 . ND3 nad3.fas +

Pphae_mito 9876 9944 62.79 6.257E-11 UCG R Metazoa_R.cm +

Pphae_mito 9945 10238 171 9.76e-51 . ND4L nad4l.fas +

Pphae_mito 10286 11530 470 6.08e-149 . ND4 nad4.fas +

Pphae_mito 11613 11682 42.42 9.573E-8 GUG H Metazoa_H.cm +

Pphae_mito 11683 11748 46.34 1.247E-7 GCU S1 Metazoa_S1.cm +

Pphae_mito 11748 11818 84.64 3.398E-18 UAG L1 Metazoa_L1.cm +

Pphae_mito 11903 13627 711 0.0 . ND5 nad5.fas +

Pphae_mito 13652 14791 647 0.0 . CYTB cob.fas +

Pphae_mito 14798 14867 62.66 1.051E-11 UGU T Metazoa_T.cm +

Pphae_mito 14882 14951 64.84 3.922E-12 UGG P Metazoa_P.cm -

Pphae_mito 14994 15431 158 5.32e-45 . ND6 nad6.fas -

Pphae_mito 15495 15567 48.42 3.505E-9 UUC E Metazoa_E.cm -

Pphae_mito 15568 17315 . . . cr Control Region +

Pphae_mito 16609 17145 1038 . . repeat Repeat Region + 3.9 copies 138 bp consensus
